# Effects of the 2018 European summer heatwave on the incidence of sporadic bacterial gastroenteritis in a temperate maritime climate region (Republic of Ireland)

**DOI:** 10.1016/j.onehlt.2025.101105

**Published:** 2025-06-10

**Authors:** Martin Boudou, Patricia Garvey, Coilín ÓhAiseadha, Jean O'Dwyer, Daniel T. Burke, Paul Hynds

**Affiliations:** a Health Information and Equality Authority, Dublin, Ireland; b Health Protection Surveillance Centre, Dublin, Ireland; c Department of Public Health, Health Service Executive (HSE), Dr. Steevens' Hospital, Dublin, Ireland; d School of Biological, Earth and Environmental Sciences, Environmental Research Institute (ERI), University College Cork, Cork, Ireland; e Environmental Sustainability & Health Institute (ESHI), Dublin, Ireland

**Keywords:** Epidemiology, Climate change, Extreme weather, Heatwave, STEC enteritis, Campylobacteriosis, Interrupted time-series analyses

## Abstract

**Background:**

The IPCC recognises that climate change and associated extreme weather events (EWEs), including heatwaves, will incur negative human health impacts. In Ireland, projections indicate more frequent and severe EWEs, however, research on the health impacts of heatwaves in temperate regions is still in its infancy.

**Purpose/aims/objectives:**

We aimed to analyse the spatiotemporal characteristics of two bacterial infections (STEC enteritis and campylobacteriosis) to quantify the public health effects of the 2018 European summer heatwave in Ireland.

**Materials and methods:**

Additive decomposition and Interrupted Time-Series Analyses (ITSA) were used to quantify effects. Excess weekly cases were calculated based on pre/post-event conditions across several case delineations (e.g., gender, age, serotype and settlement pattern.

**Results:**

Findings suggest that the summer 2018 heatwave was responsible for a minimum of 169 excess case notifications of campylobacteriosis (*n* = 101) and STEC enteritis (*n* = 68). Heatwave effects were immediate (i.e., within 10 days, *p* = 0.0019) on campylobacteriosis cases. Significant increases were observed among males, younger adults, and urban residents. STEC enteritis notification did not immediately change, but lagged effects (≥4 weeks, *p* < 0.001) were noted, with significant case notification increases identified among older adults (>65 years) and rural dwellers, alongside a notable increase in STEC O26 cases (*p* = 0.026).

**Conclusions:**

Conditions during the 2018 European summer heatwave facilitated increased case numbers of STEC enteritis and campylobacteriosis across Ireland, in concurrence with shifting demographic and geographic transmission patterns. With climate change likely favouring bacterial proliferation, further increases and pattern shifts are expected.

## Highlights

• First Irish study of the human health effects of 2018 European heatwave.
• Findings suggest heatwave responsible for minimum of 169 excess cases of campylobacteriosis (*n* = 101) and STEC (*n* = 68).
• Campylobacteriosis increased among males, younger adults, and urban residents.
• STEC enteritis increased among older adults (>65 years) and rural dwellers.

## 1 Introduction

The impact of recent west-central European heatwaves on society and nature [[1], [2], [3], [4], [5], [6]] has once again triggered questions regarding the role of climate change on the occurrence and extremity of heatwave events [[7], [8], [9], [10]]. Likewise, the association between meteorological events and human health outcomes has become increasingly apparent in recent years, with the impacts of climatological change expected to alter disease epidemiology significantly [6,11,12]. The One Health approach provides a framework to assess associations between meteorological events and the well-being of people, animals and environments and address the collective need for clean water, energy and air, safe and nutritious food, and taking action on climate change [13,14]. Global climate change is projected to generate a significantly increased burden of diarrhoeal disease, particularly via effects on hydrological and food production systems and subsequent transmission of foodborne and waterborne enteric pathogens [[15], [16], [17]]. While these projected impacts are substantial, uncertainties remain, primarily due to a sparsity of empirical climate-health data [18,19]. While numerous studies have elucidated transmission mechanisms linking diarrheal infections to heavy rainfall or flooding, few studies have quantified the associations between human health and heatwaves, particularly in temperate regions [16,19].

*Campylobacter* spp. and Shiga toxin-producing *Escherichia coli* (STEC) are two bacterial enteric pathogens with the highest global disease burden [20]. *Campylobacter* spp. is the most frequent cause of bacterial gastroenteritis across high-income regions, with an estimated 166 million infections globally per year [20,21]. STEC is estimated to cause 2.8 million acute illnesses annually, thus making it one of the most common causes of gastrointestinal illness worldwide [22]. STEC also has the capacity to cause severe renal disease, primarily in children aged under five years via haemolytic uraemic syndrome (HUS) [20,23] and is most likely to carry an increased burden of disease via climate-related changes to the hydrological cycle [20,23,24].

Studies have demonstrated that both temperature and precipitation have been correlated with the incidence of foodborne bacterial infections, including campylobacteriosis in the range of 10–25 °C [25]. Increased temperatures and warmer climactic conditions have been shown to pose a greater risk for infectious diarrhoea; for example, STEC in children has been detected in Lombardy, northern Italy, during heatwaves [[26], [27], [28]]. However, the effects of temperature and precipitation are often non-linear, and the effects of heavy rainfall are magnified after dry periods and thus warrant more study [29].

STEC proliferate in the ruminant hindgut and are spread via contact with animals, environmental surfaces, water, foodstuffs (e.g., undercooked meats), and person-to-person contact, with waterborne transmission routes well-established, particularly in areas characterised by high livestock densities and private groundwater reliance [22,23,30,31]. Foodborne STEC outbreaks in the United Kingdom have been shown to occur via direct and indirect contact with animal faecal matter or consumption of contaminated food, with person-to-person transmission in households and childcare also reported [23].

*Campylobacter* spp. is regarded as primarily foodborne, with poultry meat being implicated as the major source, along with dairy products and beef. Non-foodborne transmission routes include contact with contaminated surface waters, wildlife, pets (e.g., dogs), ruminant livestock, and person-to-person spread and potential dissemination by wind and flying insects [32]. The general environment typically serves as a vehicle for transmitting *Campylobacter* ssp. rather than as a reservoir or source as it rarely reproduces outside the gut of a warm-blooded host animal [32]. *Campylobacter* is sensitive to drying [33], making airborne transmission unlikely; however, water and soils are likely to be contaminated [32]. The use of contaminated wastewater for irrigation due to increasing water scarcity has been identified as a potential source of an increased burden of climate-related foodborne disease [34]. The association between temperature and incidence of campylobacteriosis may vary by region [35]. For example, the influence of seasonality was found to vary by region within England and Wales, being strongest in Wales and Northeast England and delayed in large urban areas (i.e., London and Birmingham) [36].

The Health Protection Surveillance Centre in Ireland maintains a series of highly validated, multi-annual datasets of statutorily notifiable diseases, including *Campylobacter* and STEC, which can be anonymised by geocoding to geographical areas representing groups of 80–120 households [37], for high-resolution spatiotemporal investigation of infectious disease. Examining the incidence profile of sporadic campylobacteriosis and STEC infection during and immediately following a heatwave period represents an opportunity to investigate and compare environmental determinants, identify high-risk populations, and distinguish differences in potential transmission pathways during heatwave periods. The present study, therefore, aimed to analyse the spatiotemporal characteristics of these two bacterial infections and quantify the effects of the 2018 European summer heatwave on public health in Ireland.

## 2 Methods

### 2.1 The 2018 European heatwave

The timeline of the 2018 west-central European summer heatwave was derived based on meteorological reports (i.e., Met Éireann and the Standardised Precipitation Index) in concurrence with numerous studies analysing the 2018 heatwave event [[38], [39], [40]]. In brief, dry and settled weather from the end of May 2018 continued through most of June and July. Slow moving anticyclones positioned themselves either directly over Ireland, just to the north, or over Scandinavia. This prolonged settled spell of weather led to heatwave and drought conditions in many parts of Ireland. Heatwave conditions were recorded at 15 synoptic stations for 5 or more days between early June and early July. Oak Park, Co Carlow recorded heatwave conditions for 11 consecutive days. During this period, Shannon Airport, Co Clare, reached 32.0 °C, the highest temperature ever recorded at a synoptic station in Ireland. The 2018 summer heatwave garnered significant media attention [39,40] and had far-reaching effects on agriculture, water supply, and water quality [38,41,42].

### 2.2 Infection data

Infection data comprised irreversibly anonymised information on cases of laboratory-confirmed STEC enteritis and campylobacteriosis reported in Ireland, collated and acquired from the national Computerised Infectious Disease Reporting (CIDR) database. Cases were geo-linked to one of 18,648 CSO Census Small Areas, the smallest administrative unit defined in Ireland. Only georeferenced primary cases of infection were included for analysis to examine the effects of the 2018 heatwave on sporadic (i.e., non-outbreak) infection. Overall, 6277 cases of STEC (84.4 % of total successfully georeferenced) were reported from January 2013 to December 2022, and 14,394 cases of campylobacteriosis (70.2 % of total) were notified from January 2011 to December 2018.

**Fig. 1:**
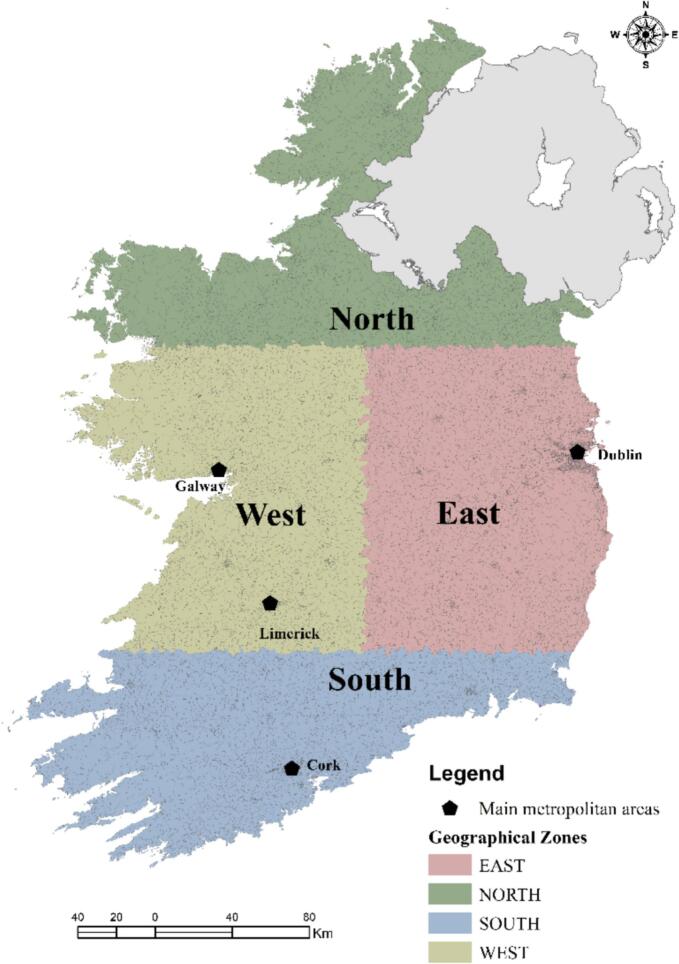
Geographical zonation based on CSO 2016 Small Areas (*N* = 18,641).

The effect of the heatwave event with respect to case gender (i.e., male/female) and delineated age groups (i.e., ≤ 5 years, 5 to 20 years, 21 to 45 years, 45 to 65 years, ≥65 years) were examined for both infections. Additionally, the impact of the heatwave on the primary STEC serotypes, specifically O26 and O157, was assessed. To account for potential spatial variation of heatwave impacts, interrupted time-series analyses were performed within each settlement type (i.e., rural, urban, and commuter areas), derived from the (Irish) Central Statistics Office (CSO) 2016 Census classification [43] and for four geographical zones. Geographical zonation was developed using the “Fishnet” tool in ArcMap v. 10.6. In cases where a Small Area was divided between two zones, the zone with the larger proportion within the Small Area was selected for analysis. The distribution of Small Areas among the four geographical zones is presented as follows (Fig. 1):

• East: 9017 (48.4 %)
• North: 2440 (13.1 %)
• West: 3191 (17.2 %)
• South: 3993 (21.4 %)

### 2.3 Seasonal adjustment and interrupted time-series analyses

Prior to statistical modelling, seasonal decomposition was conducted by applying the Seasonal and Trend (STL) decomposition method using LOESS (Locally Estimated Scatterplot Smoothing) [44,45]. The STL method decomposes the time-series of infection incidence into three distinct component series (i.e., seasonal variation, overall trend over time, and residuals), whereby the initial time-series equals the sum of these three components. Seasonally adjusted time-series, corresponding to the overall sum of trend and residuals, were extracted for each infection sub-category (i.e., clinical and geographical attributes) to examine the effect of the heatwave event. This permits removal of the annual seasonal pattern of infections, thus focusing on the direct impact of the (heatwave) event of interest on infection rates.

In the current study, ITSA was used to examine the (potential) impact of the 2018 Summer heatwave on STEC and campylobacteriosis incidence rates via the “itsa.model” function in RStudio (“its.analysis” package, version 1.6.0). This function performs a Type-2 Sum of Squares ANCOVA on a lagged dependent variable model, enabling statistical evaluation of mean differences between specific time periods [46]. The model produces several outputs, including a trimmed median F-value, used to derive bootstrapped *p*-values for both the non-lagged and lagged dependent variables. The median slope was also employed to assess the interruption effect immediately and after the 2018 Summer heatwave event. In the context of the present study, bootstrapped models with 250 iterations and a significance level α = 0.05 were employed.

### 2.4 Geostatistical analyses

Spatial autocorrelation using the Getis-Ord Gi* geostatistical tool in ArcGIS (v 10.6) was undertaken to identify potential hotspots of both infections during the 2018 summer heatwave event. The Getis-Ord GI* statistic, initially introduced by Getis and Ord [47], enables the detection of spatial clusters distinguished by notably low or high degrees of correlation. A simplified representation of the Gi* statistical formula can be expressed as follows:

$$G_{i^{*}}=\frac{\sum_{j=1}^{n}W_{\mathrm{ij}}X_{j}}{\sum_{j=1}^{n}X_{j}}$$

Where “Gi” denotes the spatial interdependence of an incident “i” with respect to all occurrences “n.” “Xj” represents the value of X at the location of the incident “j” in relation to all occurrences “n.”, and “Wij” signifies the spatial connection or weighting value between the incidents “i” and “j” [48]. This operation was performed for the entire time-series of both infections (i.e., incidence rate per 100,000 k was used to account for variability in population densities across SAs) and for the 2018 Summer heatwave period (i.e., May 22nd 2018 to July 28th 2018).

### 2.5 Ethical statement

The study was reviewed and approved by the Royal College of Physicians of Ireland Research Ethics Committee (RECSAF_84), with written informed consent waived based on the retrospective study design.

## 3 Results

### 3.1 Infection time-series

**Fig. 2:**
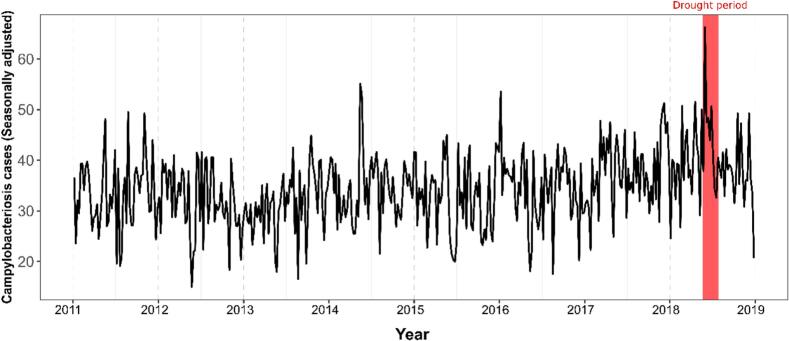
Seasonally adjusted time-series of campylobacteriosis incidence in the Republic of Ireland (2011–2018).

**Fig. 3:**
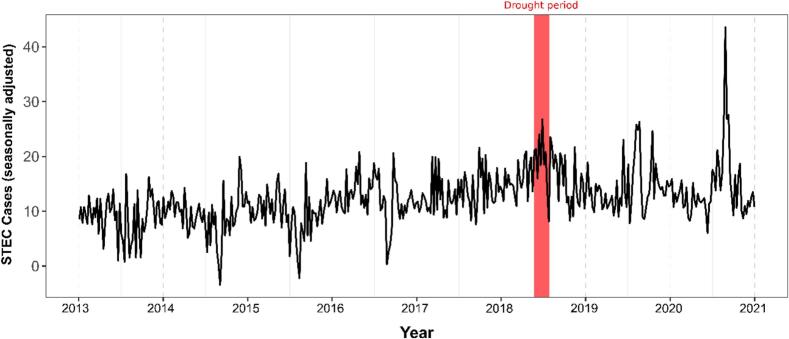
Seasonally adjusted time-series of sporadic STEC incidence in the Republic of Ireland (2013−2020).

As shown (Fig. 2), the 2018 Summer heatwave period coincided with the highest seasonally-adjusted peak of campylobacteriosis (i.e., sum of trend and residuals), e.g., +66 cases were reported during the first week of June 2018. Secondary peaks of infection are noticeable during May 2014 (+55 cases) and July 2016 (+53 cases). The highest STEC enteritis notification peak was recorded in August 2020, with approximately +43 cases (Fig. 3). June 2018, during the delineated heatwave period, registered the second highest peak over the study period, with approximately +27 cases reported during the last week of this month (Fig. 3).

Overall, 548 cases of campylobacteriosis (4.8 % of the total) and 201 cases of STEC (3.2 % of the total) were reported during the heatwave period (Table 1). Gender delineations revealed a higher proportion of STEC cases among males (51.7 %), while a higher number of female cases was noted across the rest of the time series. Similarly, a slightly higher number of male cases of campylobacteriosis was observed during the heatwave period (i.e., 57.3 %, +3.4 %). A lower percentage of both campylobacteriosis and STEC cases were noted among the <5 years and 45 to 65 age groups during the heatwave period. Conversely, slightly higher incidence rates were reported among the 21 to 45 years (STEC: +3.3 %; campylobacteriosis: +1.4 %) and > 65 years cohorts (STEC: +1.1 %; campylobacteriosis: +3.4 %). Campylobacteriosis cases were more frequently associated with urban areas across both time periods (i.e., 66.7 % and 64.8 % pre/post and during the heatwave event, respectively). Several differences were found with respect to the spatial distribution of cases across delineated geographical zones; an increase in campylobacteriosis was noted during the heatwave period in the Northern (+2.9 %) and Western (+8 %) zones, while a slight increase in STEC cases was observed in the East (+3.6 %) and North (+ 2.6 %). The proportion of STEC enteritis attributed to the O26 serotype was significantly higher during the heatwave period, with 30.8 % of total cases, compared to 24.2 % outside of the heatwave period.

**Table 1:** Infection time-series characteristics including case gender, age range, settlement type, geographical zonation and STEC serotype.

	Campylobacteriosis		STEC	
	Pre/Post heatwave period	Heatwave period	Pre/Post heatwave period	Heatwave period
Gender				
Female	6379 (46.1)	234 (42.7)	3182 (50.7)	97 (48.3)
Male	7466 (53.9)	314 (57.3)	2894 (46.1)	104 (51.7)
Age range				
<5	3188 (23.7)	90 (16.4)	2163 (35.6)	63 (31.3)
5 to 20	1936 (14.6)	80 (14.6)	966 (15.9)	34 (16.9)
21 to 45	4218 (31.9)	193 (35.2)	1002 (16.5)	36 (17.9)
45 to 65	2598 (19.5)	101 (18.4)	819 (13.5)	24 (11.9)
65+	1884 (14.2)	84 (15.3)	1125 (18.5)	44 (21.9)
Settlement type				
Rural	2623 (18.9)	118 (21.5)	,2315 (38.1)	81 (40.3)
Urban	9237 (66.7)	355 (64.8)	2258 (37.2)	74 (36.8)
Commuter	1985 (14.3)	75 (13.7)	1503 (24.7)	46 (22.9)
Geographical Zone				
East	7024 (50.7)	262 (47.8)	2117 (34.8)	75 (37.3)
North	1195 (8.6)	63 (11.5)	708 (11.7)	28 (13.9)
West	2251 (16.3)	133 (24.3)	1641 (27)	54 (26.9)
South	3315 (23.9)	90 (16.4)	1610 (26.5)	44 (21.9)
Serotype (STEC)				
O26	–	–	1522 (24.2)	62 (30.8)
O157	–	–	1127 (18)	32 (15.9)
Total				
	13,845 (96.2)	548 (4.8)	6076 (96.8)	201 (3.2)

### 3.2 Interrupted time-series analysis

#### 3.2.1 Campylobacteriosis

The heatwave period was marked by an increase in the incidence rate of campylobacteriosis, with +10.08 mean weekly cases reported during this period (p-value: <0.001) (Table 2). All age groups apart from the <5 years old cohort exhibited an increase, all of which were statistically significant apart from the 45- to 65-years cohort. The largest increase was noted among males, with a mean weekly incidence of 25.5 cases during the heatwave period, corresponding to an increase of 7.1 cases/week. The 21–45 age range reported the highest increase with +5.62 mean weekly cases. Campylobacteriosis cases associated with urban and rural areas were linked with increases of +6.09 cases and + 2.91 mean cases per week, respectively. Both Northern and Southern geographical areas reported a relatively similar increase in campylobacteriosis incidence during the heatwave period, with associated mean incidence rates of 21.2 (+2.43) and 10.6 (+2.47) mean weekly cases, respectively.

**Table 2:** Results of interrupted time-series analyses for campylobacteriosis.

	Sum of squares	F Value	P-value	Mean weekly incidence prior/post heatwave	Mean weekly incidence heatwave	Difference heatwave/non-heatwave periods
Gender						
Female	63.90	4.109	0.0433*	15.7	18.8	**+3.04**
Male	283.20	10.739	0.0011*	18.4	25.5	**+7.10**
Age groups						
<5	12.00	1.359	0.244	7.8	6.6	−1.22
5 to 20	27.82	5.441	0.0201*	4.8	6.6	**+1.86**
21 to 45	250.90	19.971	<0.001*	12.0	17.6	**+5.62**
45 to 65	8.80	1.124	0.290	7.5	8.7	+1.19
65+	24.03	4.204	0.041*	4.7	7.2	**+2.55**
Settlement type						
Rural	67.63	9.773	0.0019*	6.5	9.4	**+2.91**
Urban	212.60	7.855	0.0053*	22.8	28.9	**+6.09**
Commuter	8.63	1.583	0.209	4.9	5.9	+1.09
Geographic zone						
East	67.10	3.069	0.081	17.4	21.2	+3.86
North	53.76	19.496	<0.001*	2.9	5.4	**+2.43**
West	17.71	3.293	0.070	5.6	7.1	+1.46
South	45.60	5.085	0.0247*	8.1	10.6	**+2.47**
Republic of Ireland (Total)						
	0.80	9.804	0.0019*	34.1	44.2	+10.08

#### 3.2.2 STEC enteritis

A significant national increase in STEC incidence during the heatwave period was noted (p-value: <0.001), with a mean weekly incidence of 18.9 cases/week, reflected across all infection sub-categories (Table 3). The increase was statistically significant for male cases, with approximately +3.71 cases/week during the heatwave event. A similarly significant increase was observed among the 21 to 45 years and elderly (> 65 years old) age ranges, with +1.41 and + 2.32 mean cases/week, respectively. A statistically significant elevated incidence of rural cases was identified, with approximately 6.5 cases/week during the heatwave period compared with 4.3 cases/week pre/post event (+2.24 cases/week). Likewise, Eastern and Northern regions exhibited a statistically significant increase, with +2.43 and + 1.29 mean cases/week during the heatwave period. Results for the two main STEC serotypes identified an atypical excess of serotype O26 cases with a mean of 4.7 cases/week from May to July 2018 (+1.53).

**Table 3:** Results of the interrupted time-series analysis for STEC.

	Sum of squares	F Value	P-value	Mean weekly incidence prior/post heatwave	Mean weekly incidence heatwave	Difference heatwave/non-heatwave periods
Gender						
Female	30.6	3.686	0.056	6.4	9.5	+3.09
Male	63.88	9.734	0.002*	5.7	9.4	**+3.71**
Age groups						
<5	7.27	1.543	0.215	4.3	5.4	+1.01
5 to 20	6.53	2.947	0.087	1.9	3.3	+1.41
21 to 45	16.75	7.419	0.007*	2.0	3.6	**+1.55**
45 to 65	1.37	0.709	0.400	1.7	2.2	+0.51
65+	35.94	14.899	<0.001*	2.1	4.5	**+2.32**
Settlement type						
Rural	42.37	9.898	0.002*	4.3	6.5	**+2.24**
Urban	10.19	2.162	0.142	4.3	5.9	+1.66
Commuter	8.28	2.613	0.107	2.8	4.0	+1.16
Geographic zone						
East	30.23	7.927	0.005*	3.9	6.4	**2.43**
North	53.76	19.496	<0.001*	1.2	2.5	**+1.29**
West	0.15	0.048	0.827	3.1	3.2	+0.14
South	5.64	1.486	0.224	3.1	4.3	+1.19
Serotypes O26 & O157						
O26	18.37	4.974	0.026*	3.2	4.7	**+1.53**
O157	9.47	3.356	0.068	2.5	3.8	+1.28
Republic of Ireland (Total)						
	522.6	19.457	<0.001*	12.1	18.9	**+6.80**

### 3.3 Spatial autocorrelation

#### 3.3.1 Campylobacteriosis

**Fig. 4:**
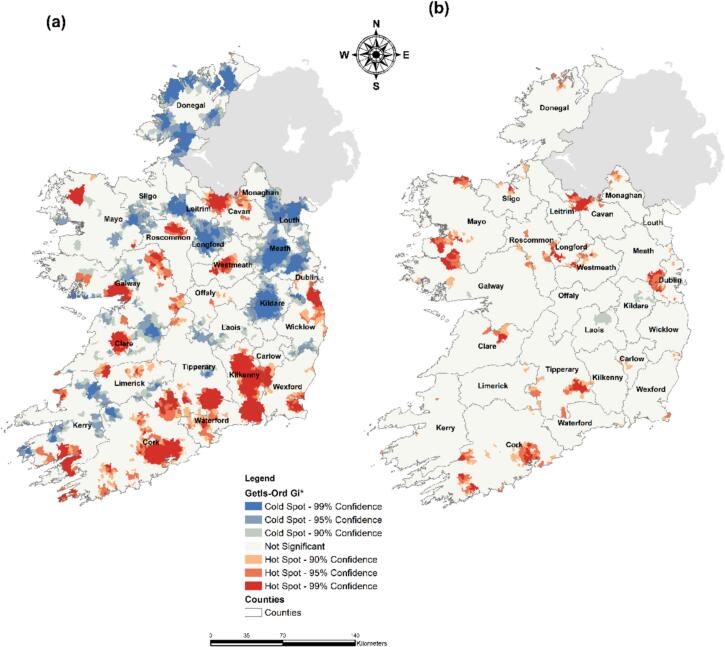
Spatial hot and cold spots of campylobacteriosis incidence rate/100 k people (Getis-Ord Gi*) – (a) Prior & post heatwave period – (b) Heatwave period.

Results for the non-heatwave period primarily identified hotspots of infections around the main city areas of Ireland (i.e., Dublin, Cork, Galway and Limerick) along with counties located in the South-East of the country (i.e., Tipperary, Kilkenny, Waterford, Wexford) (Fig. 4a). Conversely, cold spots of infection were in the northern part of the country (i.e., Donegal, Louth, Leitrim). Spatial autocorrelation for the heatwave period (Fig. 4b) highlighted slight differences regarding hotspot location. While the Cork metropolitan area remained an infection hotspot, Dublin and Galway metropolitan areas were no longer identified as such. Conversely, a new hot spot appeared in the northern part of the country, specifically counties Donegal and Sligo. Similarly, areas situated in the western part of the Dublin metropolitan area and in the southern part of county Mayo were highlighted as new infection hotspots.

#### 3.3.2 STEC enteritis

**Fig. 5:**
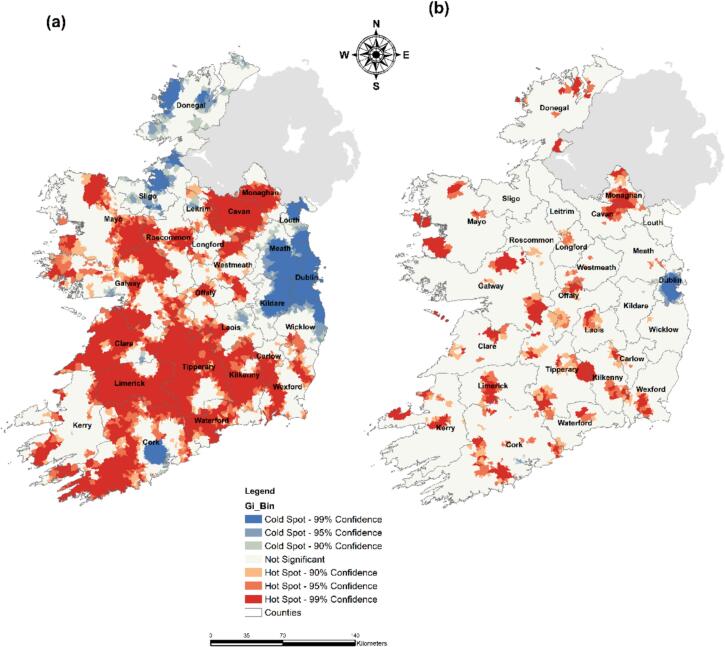
Spatial hot and cold spots of STEC incidence rate/100 k people (Getis-Ord Gi*) – (a) Prior & post heatwave period – (b) Heatwave period.

As shown (Fig. 5a), a wide distribution of STEC infection hotspots was found across the Irish midlands, on an axis from Cork (south) to Monaghan (north) counties, for the pre- and post-heatwave period (2013–2020). Infections cold spots were primarily located in Northern areas (i.e., Donegal and Sligo counties), the East Coast (i.e., Dublin, Meath, Wicklow and Louth counties), and around the Cork metropolitan area. Results for the 2018 heatwave period highlighted significant differences regarding the location of STEC infection hotspots. New infection hotspots were identified in the North (i.e., Donegal). Similarly, the East Coast was no longer identified as a cold spot of infection, apart from the Dublin metropolitan area.

## 4 Discussion

The presented research assesses the impact of the 2018 west-central European heatwave on the incidence of two common enteric bacterial infections in the ROI, a temperate maritime climatic region typically unaffected by heatwave events. Overall, findings suggest several associations between the 2018 event and both campylobacteriosis and STEC infections. While interpretation of the epidemiology of enteric pathogens is complicated by the myriad interactions between climatic, behavioural and environmental factors [49,50], the 2018 summer heatwave was shown to coincide with the highest and second highest seasonally-adjusted peaks of campylobacteriosis and STEC infections for that year, respectively. In Ireland, disease outbreaks are often preceded by heavy precipitation [50,51], initiated by surface runoff of pathogens to private drinking water resources [52]. However, presented findings suggest that the incidence of infection is also influenced by periods of markedly reduced rainfall (i.e., heatwaves and drought), as evidenced elsewhere. For example, a recent study by Brehm et al. (2021) reported that heatwaves during the summer of 2018 and 2019 were responsible for at least 63 *Vibrio* spp. infections adjacent to the German Baltic Sea and its estuaries [19]. In the present study, however, the evidence is unclear as to the role of drinking water as a vector for disease transmission.

Urban areas were associated with an increased burden of disease during the heatwave period (+6.09), and thus, contaminated drinking water is an unlikely transmission route due to the water infrastructure in Ireland (all urban areas are served by publicly managed, chemically treated water supplies). Rather, the increased incidence of the disease in urban areas is likely attributable to the daily temperatures noted at the time of the heatwave; increases in sunlight hours and warmer temperatures have a direct effect on human activities that increase exposure to *Campylobacter* spp. sources [19,53]. Known risk factors including contact with farm animals [30,54], swimming, camping, barbecuing, and other outdoor recreation [19,55,56] have been linked with an increased risk of infection. The exacerbation of heat-related behaviours by higher temperatures is further supported by an increased incidence in infection rates noted among 21- to 45-year-olds (+5.62), who are most likely to engage in heat-related activities, where exposure risk increases (e.g. outdoor swimming, barbeques, etc.). Similarly, Sanderson et al. (2018) have suggested that an increased incidence of human campylobacteriosis in the Tyne catchment (northern England) during the summer may be a consequence of increased outdoor activities, transmission by wildlife and an increased risk of consumption of partially cooked meat from barbeques [57].

Semenza et al. (2012) identified fresh vegetables, meat, and raw milk as foods that can easily be contaminated with *Campylobacter*, leading to faecal-oral transmission [58]. The easily contaminated fresh vegetables, meat, and raw milk raise the hypothesis that irrigation with contaminated water may have contributed to the increased incidence, as traditionally ‘rain fed’ crops were irrigated with nearby surface waters, where feasible, during the heatwave period [59]. Moreover, the present study found younger cohorts, 5–20 (*p* = 0.02) and 21–45 (*p* < 0.001) years, to have significantly more cases during the heatwave period by +1.86 and + 5.62, respectively, when compared to the non-heatwave period. This is in line with other studies where effects of temperature on the incidence of campylobacteriosis have been shown to vary by age group. For example, temperature had a greater influence on infection among young children (0 to 10 years), and infection increased for ages 0 to 34 and 16 to 34 years close to high ruminant density and poultry operations, respectively [60,61].

The incidence of STEC infections also increased (+6.8) during the heatwave. A statistical association was noted between STEC cases and categorically rural areas, which is consistent with infection via a variety of previously documented routes of transmission, primarily waterborne [24,31,62]. However, it should be noted that heat-related behaviours will also have played a role, with direct animal contact and consumption of improperly cooked meats (e.g., barbeques) also representing key likely risk factors. For example, in the United States, improperly cooked ground beef is the most common food associated with STEC infections [63]. In Ireland, STEC cases in rural areas have been consistently linked with the use of private groundwater sources, with microbiological contamination (*E. coli*) of groundwater reported in 66.7 % of monitoring wells (*n* = 132) over a 10-year period in Ireland [45]. However, research by O'Dwyer et al. (2021), reported a decrease in private well contamination during the 2018 heatwave, with increases in contamination resuming (and amplified) following post-heatwave rainfall events i.e., enhanced mobilisation of the pathogen to groundwater supplies following resumption of “normal” hydrogeological conditions [41].

During the pre-post event periods, both campylobacteriosis and STEC cold spots were identified in northern regions; however, during the heatwave period, hot spots emerged in this region. Moreover, during the heatwave period, hot spots for campylobacteriosis and STEC developed around the Dublin metropolitan area, perhaps indicating that urban residents are more susceptible to enteric infections during heatwave conditions. These developing hot spots may result from increased exposure risk related to weather-related activities (e.g. outdoor swimming, barbeques, etc.) in concurrence with lower levels of acquired immunity than seen in those of rural residents [20]. Additionally, the average age of residents associated with cities (37 years), satellite urban towns (34.5 years), and independent towns (36.5 years) were lower than rural areas with high urban influence (37.5 years), without high urban influence (38.9 years) and highly rural areas (41.2 years) [43]. This would be consistent with the significantly higher incidence of campylobacteriosis and STEC cases during the heatwave period among the 21 to 45 age group and identified hotspots in the Dublin metropolitan area.

Agricultural produce transported from rural to urban areas during the heatwave may also be responsible for hot spot development in the Dublin metropolitan area for campylobacteriosis and STEC, as traditionally ‘rain-fed’ produce (i.e., fresh vegetables) may be irrigated or processed with contaminated water [59]. Abattoir methods for poultry and beef processing may also increase *Campylobacter* spp. infections during heatwaves [64,65]. Meat processing and transportation in Ireland may not be appropriate during heatwaves with most meat produced and consumed domestically - Ireland's meat production levels currently stand at 262 % of domestic demand [66]. Therefore, additional resources (i.e., disease monitoring, education for correct cooking methods, improved heatwave-resilient meat slaughtering and transportation methods, and enhanced agricultural quality control) may be necessary for heatwave events, especially with heatwave frequency predicted to increase due to climate change [67,68]. STEC hot spots were found in more rural and agriculturally intense areas (axis from Cork to Monaghan County), mirroring current literature as the transmission of STEC is primarily waterborne and associated with private groundwater sources in rural areas [24,31,62]. Under the One Health approach, improving livestock and agricultural practices, such as reducing livestock density, adjusting farming cycles to align with a changing climate, and providing animals with longer shelter during wetter seasons, may not only enhance animal health but also reduce STEC and *Campylobacter* spp. infections, by minimising habitat destruction caused by the expansion of livestock farming and mitigating environmental changes that disrupt the human-animal-environment interface [69]. Therefore, close interdisciplinary cooperation is essential to protect public health and align with the One Health framework; for example, an approach involving all stakeholders (e.g., veterinary authorities, medical experts, scientists, policymakers, public health officials, and the agricultural industry) is crucial to reduce the burden of STEC and *Campylobacter* spp. in humans, especially within the context of climate change [70] [71].

Overall, this research demonstrates that heatwave conditions can, directly and indirectly, increase the transmission of pathogens like *Campylobacter* spp. and STEC to humans. While foodborne and waterborne sources are considered the major transmission pathways for *Campylobacter* and STEC [72], findings reported here suggest that meteorological variability will play an increasingly significant role in contamination events, warranting further investigation. For example, climate change will likely lead to a potential increase in the threat of zoonotic foodborne illnesses, both directly and indirectly. Livestock animals are more susceptible to microbial infections due to the impact of climate change on their living conditions, making them important reservoirs for diseases. Furthermore, the proliferation of animal pests can act as vectors for zoonotic diseases. Changes in atmospheric conditions, sea temperatures, weather events, ocean acidification, and salinity levels all have implications for the health of seafood, affecting the survival and transmission capacity of human infections present in marine environments and seafood. These various pathways of foodborne illnesses are influenced by climate change, thereby compromising the quality and safety of multiple food items [73].

## 5 Conclusions

The present study suggests that the summer 2018 heatwave was directly or indirectly responsible for at least 169 excess cases of campylobacteriosis and STEC enteritis in Ireland. While this may seem a relatively modest increase at the national level, crucially, when upscaled to a European level, this equates to several thousand excess cases of seasonally and spatially atypical infection, thus representing significant extra pressure on surveillance and healthcare systems. An extreme summer such as the 2018 heatwave is expected to occur every two out of three years in Europe in a + 1.5 °C warmer world and every year in a + 2 °C warmer world [74]. With the potential for heatwave impacts amplified by population growth, urban expansion, ageing or deteriorating infrastructure, and environmental protection, a rise in these bacterial enteric infections is expected in the future.

## CRediT authorship contribution statement

**Martin Boudou:** Writing – original draft, Visualization, Methodology, Formal analysis, Data curation, Conceptualization. **Patricia Garvey:** Writing – review & editing, Resources, Data curation. **Coilín ÓhAiseadha:** Writing – review & editing, Software, Resources, Data curation. **Jean O'Dwyer:** Writing – review & editing, Supervision, Resources, Project administration, Funding acquisition, Conceptualization. **Daniel T. Burke:** Writing – review & editing. **Paul Hynds:** Writing – original draft, Validation, Supervision, Project administration, Methodology, Funding acquisition, Conceptualization.

## Declaration of competing interest

All authors confirm that no financial or personal relationships with other people or organizations exist that could inappropriately influence (bias) the submitted work.

## Data Availability

The authors do not have permission to share data.
